# Robust vs. Non-robust radiomic features: the quest for optimal machine learning models using phantom and clinical studies

**DOI:** 10.1186/s40644-025-00857-1

**Published:** 2025-03-12

**Authors:** Seyyed Ali Hosseini, Ghasem Hajianfar, Brandon Hall, Stijn Servaes, Pedro Rosa-Neto, Pardis Ghafarian, Habib Zaidi, Mohammad Reza Ay

**Affiliations:** 1 Translational Neuroimaging Laboratory, Douglas Hospital, The McGill University Research Centre for Studies in Aging, McGill University, Montréal, Québec Canada; 2 Department of Neurology and Neurosurgery, Faculty of Medicine, McGill University, Montréal, Québec Canada; 3 Division of Nuclear Medicine and Molecular Imaging, Geneva University Hospital, CH-1211 Geneva 4, Switzerland; 4 Chronic Respiratory Diseases Research Center, National Research Institute of Tuberculosis and Lung Diseases (NRITLD), Shahid Beheshti University of Medical Sciences, Tehran, Iran; 5 PET/CT and cyclotron center, Masih Daneshvari Hospital, Shahid Beheshti University of Medical Sciences, Tehran, Iran; 6 Department of Nuclear Medicine and Molecular Imaging, University of Groningen, University Medical Center Groningen, 9700 RB Groningen, Netherlands; 7 Department of Nuclear Medicine, University of Southern Denmark, DK-500 Odense, Denmark; 8 University Research and Innovation Center, Óbuda University, Budapest, Hungary; 9 Department of Medical Physics and Biomedical Engineering, Tehran University of Medical Sciences, Tehran, Iran; 10 Research Center for Molecular and Cellular Imaging (RCMCI), Advanced Medical Technologies and Equipment Institute (AMTEI), Tehran University of Medical Sciences (TUMS), Tehran, Iran

**Keywords:** PET¸ Radiomic features, NSCLC, Lymphovascular invasion, Feature selection, Machine learning, Robustness, Motion artifacts

## Abstract

**Purpose:**

This study aimed to select robust features against lung motion in a phantom study and use them as input to feature selection algorithms and machine learning classifiers in a clinical study to predict the lymphovascular invasion (LVI) of non-small cell lung cancer (NSCLC). The results of robust features were also compared with conventional techniques without considering the robustness of radiomic features.

**Methods:**

An in-house developed lung phantom was developed with two 22mm lesion sizes based on a clinical study. A specific motor was built to simulate motion in two orthogonal directions. Lesions of both clinical and phantom studies were segmented using a Fuzzy C-means-based segmentation algorithm. After inducing motion and extracting 105 radiomic features in 4 feature sets, including shape, first-, second-, and higher-order statistics features from each region of interest (ROI) of the phantom image, statistical analyses were performed to select robust features against motion. Subsequently, these robust features and a total of 105 radiomic features were extracted from 126 clinical data. Various feature selection (FS) and multiple machine learning (ML) classifiers were implemented to predict the LVI of NSCLC, followed by comparing the results of predicting LVI using robust features with common conventional techniques not considering the robustness of radiomic features.

**Results:**

Our results demonstrated that selecting robust features as input to FS algorithms and ML classifiers surges the sensitivity, which has a gentle negative effect on the accuracy and the area under the curve (AUC) of predictions compared with commonly used methods in 12 of 15 outcomes. The top performance of the LVI prediction was achieved by the NB classifier and RFE FS without considering the robustness of radiomic features with 95% area under the curve of AUC, 67% accuracy, and 100% sensitivity. Moreover, the top performance of the LVI prediction using robust features belonged to the NB classifier and Boruta feature selection with 92% AUC, 86% accuracy, and 100% sensitivity.

**Conclusion:**

Robustness over various influential factors is critical and should be considered in a radiomic study. Selecting robust features is a solution to overcome the low reproducibility of radiomic features. Although setting robust features against motion in a phantom study has a minor negative impact on the accuracy and AUC of LVI prediction, it boosts the sensitivity of prediction to a large extent.

## Introduction

The occurrence of lung cancer and related deaths have risen in the past decade, owing in part to pollution, smoking rates, and advances in diagnosis [1]. In 2018, more than 1.76 million fatalities and 2 million new cases were predicted by the Global Cancer Observatory Organization (GLOBOCAN), considerably higher than the 2012 statistics (1.6 million deaths and 1.8 million new cases) [2]. Lung cancer is the second most common cancer in both sexes (after breast cancer in women and prostate cancer in men) and it has the highest mortality rate [3]. Recently, there have been significant breakthroughs in the treatment of non-small cell lung cancer (NSCLC), including immunotherapy, chemotherapy, and molecular-targeted therapy [4]. However, NSCLC cure and survival rates are still poor, especially in metastatic illnesses [4], due to a number of limitations, including late-stage diagnosis, treatment resistance, or recurrence.

In resected non-small cell lung carcinoma (NSCLC), lymphovascular invasion (LVI) is regarded as a high-risk pathologic characteristic [5]. The ability to divide stage I patients into risk categories may allow adjuvant therapy recommendations to be refined [6]. Towards that end, we present a technique that utilizes advanced image analysis of positron emission tomography (PET)/computed tomography (CT) imaging. PET is a molecular medical imaging modality widely used to detect early signs of cancer, brain disorders, and heart disease. Combining PET with CT in a concurrent acquisition produces 3D images that are superior to PET and CT images acquired separately [7].

Radiomics is an emerging quantitative technique designed to extract analyzable data from multimodality medical imaging modalities [8, 9]. The application of radiomics in medicine has been widely reported along with machine/deep learning for predicting [10], diagnosing [11] abnormalities, and predicting response to therapy [12]. Conversely, NSCLC presents a complex imaging scenario due to the lungs' inherent motion and the tumor's proximity to moving structures [13]. The high variability in tumor size, location, and the surrounding lung parenchyma further complicates image analysis and feature extraction. These factors underscore the necessity for precise and robust radiomic analysis capable of accommodating or correcting for motion-induced variability [14]. Motion artifacts introduce blurring and distortions in imaging, thus affecting the accuracy of quantitative radiomic features. For instance, texture features, crucial for discriminating between benign and malignant lesions or for predicting gene expression profiles, can be altered by the blurring effect of motion, leading to potential misclassification or incorrect prognostic assessment [13].

During the recent decade, advances in medical imaging technology has been immense, leading to significant improvement in image quality and quantitative accuracy [15]. However, medical images are still vulnerable to various factors that may affect quantitative imaging [16]. This might impact machine learning and deep learning radiomic studies [17, 18]. Moreover, the repeatability and reproducibility of radiomic features have always been under scrutiny [19]. Previous studies indicated that several factors may impact medical images qualitatively and quantitatively and that various factors may affect radiomic features, such as image reconstruction [13], pre-processing [19], respiratory motion [13], image acquisition [14], segmentation techniques [17], and test-retest [13]. Among these factors, respiratory motion and the use of multi-centric images may especially impact the radiomic features to a large extent [13]. Motion artifacts exacerbate this challenge, as features that are not robust against such artifacts may show considerable variability, undermining their predictive power and clinical utility. Therefore, the need for features that maintain their integrity and predictive capability, despite the presence of motion artifacts, is paramount [20].

Previous research in quantitative analysis has offered possible solutions to tackle this problem, including selecting robust features against effective factors [21] and ComBat harmonization [22]. Although selecting robust features against influential factors has been widely examined in previous clinical [23] and phantom [24] studies, the application of robust features in the clinic has been overlooked, and in the majority of previous studies, features were selected by feature selection algorithms [25]. In the realm of machine learning, feature selection algorithms are essential for enhancing model efficiency and interpretability by selecting the most influential features from large datasets. These algorithms streamline model training, mitigate overfitting, and facilitate faster computational processes, which is particularly advantageous in fields burdened with high-dimensional data, such as multimodality medical imaging. However, their application is not without challenges; they may overlook the interaction among features and the robustness across varied datasets, which can lead to models that perform well under specific conditions but falter more broadly. As such, while feature selection can significantly refine the predictive power and clarity of machine learning models, it is crucial to validate the chosen features’ performance across diverse scenarios to ensure their reliability and applicability.

Among radiomics machine learning studies, feature selection algorithms like Boruta, LASSO, Recursive Feature Elimination (RFE), Minimum-Redundancy-Maximum-Relevance (MRMR), and even selecting features using classifiers, such as Random Forest, are employed to identify the most relevant features from a high-dimensional dataset [26], usually focusing on those with strong predictive power, often termed as "bold" features. These algorithms prioritize features that provide the maximum discriminatory or predictive power for the outcome of interest, such as disease classification [27]. However, they may overlook robust features that remain stable across different imaging protocols or conditions, as the algorithms are generally optimized for maximum performance on a specific dataset rather than generalizability [27].

Given these challenges, our study aims to investigate the impact of motion artifacts on the predictive accuracy of radiomic features in NSCLC, with a particular focus on identifying and evaluating the robustness of features against motion, followed by another round of feature selection to predict LVI in NSCLC. By doing so, we aim to enhance the reliability of radiomics as a tool for the early detection, characterization, and treatment planning of NSCLC, ultimately contributing to improved patient outcomes.

## Materials and methods

**Fig 1:**
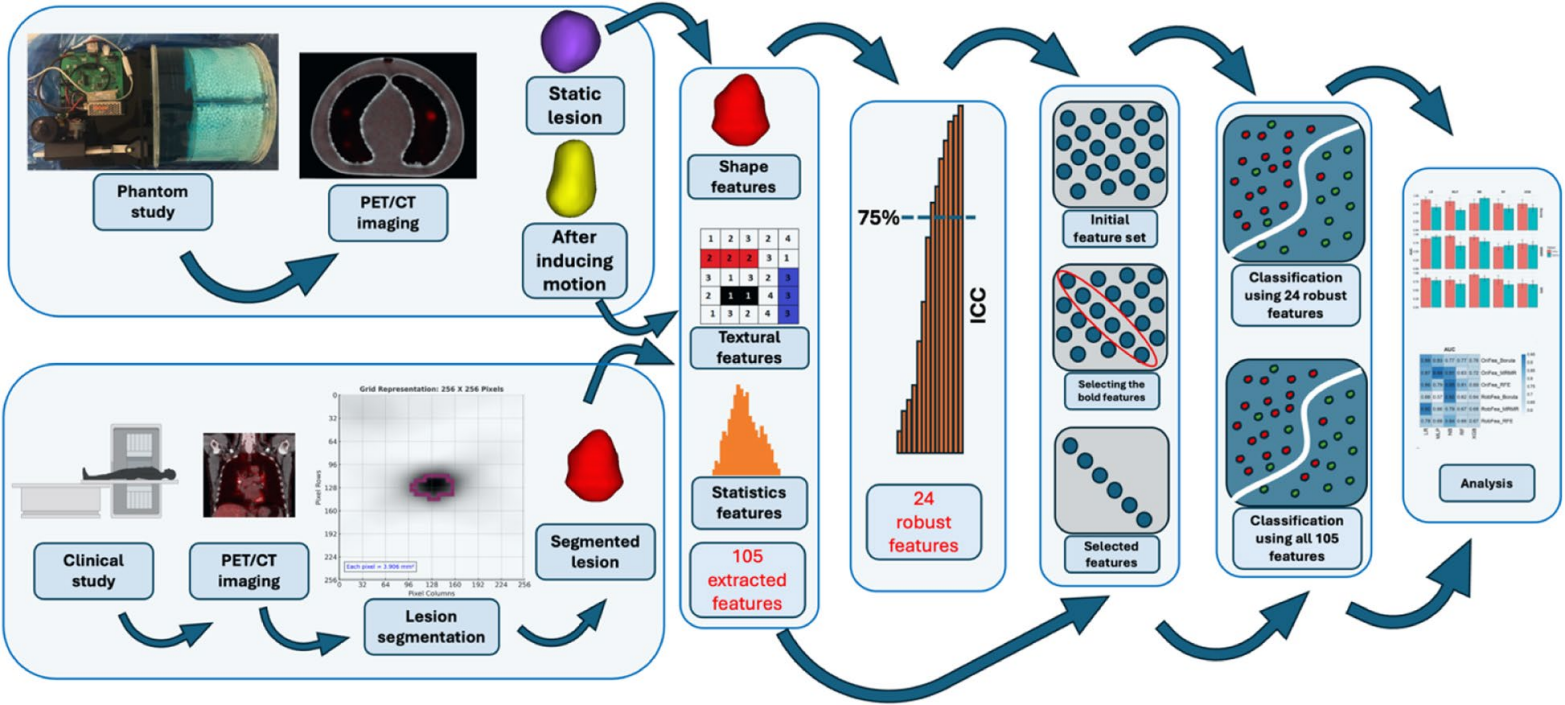
The framework implemented in this study starts with the induction of motion, clinical study, proceeds through image processing and feature extraction, and concludes with data analysis.

The framework implemented in the current study is depicted in Figure 1.

### Phantom study

A GE Discovery 690 PET/CT scanner (General Electric Healthcare, USA) was used for the phantom study. In this investigation, an in-house thoracic phantom was constructed with two spherical inserts with inner diameters of 22 mm (left and right), 9.6 L capacity, and 180 mm interior length. To eliminate partial volume effect (PVE) in a realistic phantom investigation, all lesions did not include walls.

**Fig 2.**
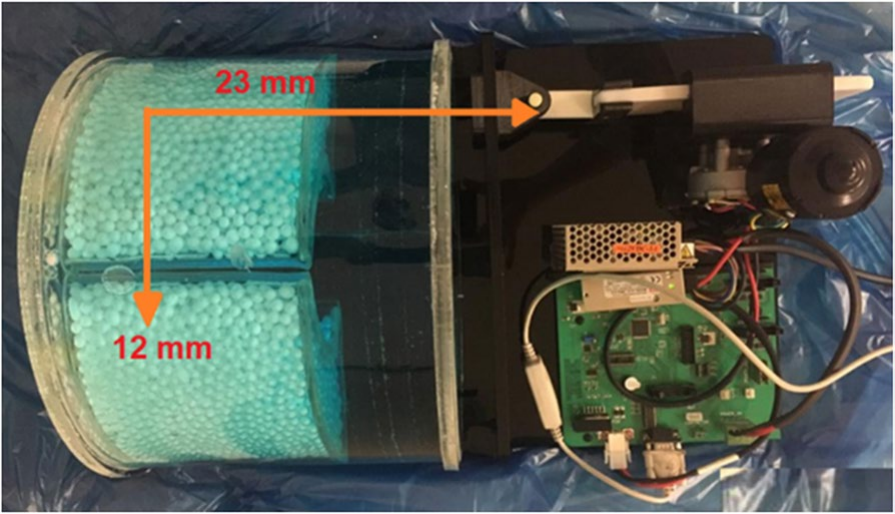
In-house developed thoracic phantom and motor for simulating lung motion (13).

We built a motor that was put beneath the phantom to induce breathing movements. This motor caused lung motion (in two orthogonal directions: lateral and posterior-anterior) at a rate of 12 breaths per minute, which is the average respiratory rate of a healthy adult at rest [28]. The thoraco-abdominal lesion moves between 6 and 23 mm in each direction due to respiratory motion [29], closely mimicking clinical scenarios for a normal subject. We induced 12 mm in one direction and 23 mm in the other, 12 times in one minute. The phantom and lesions were filled with a combination of ^18^F-FDG and water with activity concentrations of 5.3 kBq/ml and 2.65 KBq/ml, respectively, corresponding to 370 MBq and 185 MBq injected to a 75 kg patient, respectively (Figure 2). The outcome was a 256 × 256 image grid, where each pixel covered a 3.906 mm^2^ area. To refine the image, a Gaussian post-processing filter with 4.5 mm full width at half maximum (FWHM) was utilized. To reduce the impact of test-retest on our results, the scanning was repeated three times, with and without inducing motion and averaged over 3 repetitive times.

### Clinical studies

The Institutional Review Board (IRB) of Tehran University of Medical Sciences granted approval for this retrospective study, under approval ID IR.TUMS.MEDICINE.REC.1397.733. Given the study's retrospective design, the IRB waived the need for written informed consent from patients. Data of 126 patients (72 males and 54 females; mean age: 48 ± 11) were collected between March 2019 and January 2022. Each patient included in the study was diagnosed with NSCLC through biopsy and had either positive or negative LVI, resulting in an imbalanced dataset with 50 LVI positive cases and 76 LVI negative cases. Before undergoing ^18^F-FDG-PET imaging, patients were required to fast for a minimum of six hours. Therefore, prior to the scan, blood glucose levels were measured using a standard glucometer, a common medical device that provides quick and accurate glucose readings. Patients with glucose levels exceeding 200 mg/dl were rescheduled, as optimal imaging conditions necessitate glucose levels within the normal range to avoid competition between glucose and the^18^F-FDG. The PET scans were taken between 50 and 70 minutes post-injection. Imaging was performed on a GE Discovery 690 PET/CT scanner, similar to the phantom study. For anatomical mapping and attenuation correction, a low-dose CT scan was performed. PET data was reconstructed using the ordered subset-expectation maximization (OSEM) iterative technique, which involved three iterations and 18 subsets. This resulted in an image grid of 256 × 256, with each pixel spanning an area of 3.906 mm^2^. A Gaussian post-processing filter, having a FWHM of 4.5 mm, was applied. Consistency in image generation was maintained by using the same reconstruction method, subsets, and iterations to minimize variations that could affect the reliability of the imaging data. To minimize pre- and post-processing variances, all clinical and phantom images were acquired using the same reconstruction technique and the same number of subsets and iterations (Table 1).

**Table 1:** Study patients' clinical and pathological features.

**Characteristics**	
**Gender**	
Male	72
Female	54
**Height (cm) (mean±SD)**	161±21
**Weight (kg) (mean±SD)**	69±13
**Histology**	
LVI positive	50
LVI negative	76

### PET image segmentation

In clinical and phantom image analysis, MATLAB 2022a was utilized to implement a Fuzzy C-means (FCM)-based segmentation algorithm to delineate lesions and tumors [30]. FCM is a clustering algorithm that assigns each data point to one or more clusters based on its degree of membership. It generalizes the k-means algorithm by allowing soft assignment rather than hard assignment of points to clusters [31]. In image segmentation, it is commonly used to partition an image into regions with similar characteristics based on pixel values. All segmentation procedures and results were controlled and validated by two PET medical physicists with 15 and 10 years of experience.

### Feature extraction

One hundred and five 3D radiomic features were extracted from each region of interest (ROI) in clinical and phantom studies using the “Image Biomarker Standardization Initiative” (IBSI) [32, 33] compliant Pyradiomics package [34] in Python. These features were categorized into four feature sets, including shape (*n*=13), first-order (*n*=18), second-order and higher-order texture (Gray Level Dependence Matrix (GLDM) (*n*=14), (Gray Level Co-occurrence Matrix (GLCM) (*n*=23), Gray Level Size Zone Matrix (GLSZM) (*n*=16), Gray Level Run Length Matrix (GLRLM) (*n*=16), and Neighboring Gray Tone Difference Matrix (NGTDM) (*n*=5).

### Feature selection

#### Robust feature section

Robust features were selected using the phantom study. After inducing motion and starting data acquisition, radiomic features were extracted from the static and simulated lung motion images. Next, the intraclass correlation of coefficient (ICC) was calculated for each radiomic feature, between the motion and static situations. Radiomic features were categorized based on their ICC into four groups: 1) 90%>ICC>100%, 2) 75%<ICC<90%, 3) 50%>ICC>75%, and 4) ICC<50%. Radiomic features with more than 75% ICC were selected as robust features. All calculations were implanted in R version 4.0.4 (The R Foundation, Vienna, Austria) using the ‘irr’ library (version 0.84.1) [35–37].

#### Feature selection algorithms

Various feature selection algorithms were implemented in the current study, including Boruta, Recursive Feature Elimination (RFE), and Minimum redundancy maximum relevance (MRMR). The dataset was divided into 70%/30% training/validation sets, with Z-score normalization applied to the training set, and with mean and standard deviation applied to the validation set, derived from the training set. The MRMR feature selection technique was used to pick a total of 10 features. We didn't employ a predefined number of selected features in REF and Boruta feature selection; instead, the technique provided the number. We applied a quantitative threshold to ensure that each selected feature had high relevance to our outcome of interest while sharing minimal information with other selected features, optimizing both predictive power and data efficiency. This methodological choice was crucial for enhancing the robustness and interpretability of our predictive model.

MRMR: This method selects features based on two criteria. The "Maximum Relevance" part aims to pick features that are highly correlated with the target variable, ensuring that the chosen features are meaningful. The "Minimum Redundancy" aspect aims to make sure that the selected features are as different from each other as possible to avoid overfitting and multicollinearity. The "Minimum Redundancy" part of MRMR could be particularly useful for radiomics, where features can often be highly correlated due to the nature of medical imaging. By ensuring that redundant features are eliminated, MRMR increases the robustness of the selected features [38].

REF: In this method, a model is trained on the initial set of features, and the least important features (often judged by their coefficients or feature importance) are eliminated. The model is then retrained with the remaining features, and the process repeats until a predefined stopping condition is met or the model performance no longer improves. REF is iterative, which means it takes into account how the removal of one feature affects the importance of others. This makes the method adaptive and potentially more robust, useful for radiomics where the interactions between features can be complex [39].

Boruta: This is a randomized feature selection method. It creates shadow features (random permutations of the original features) and trains a model like a Random Forest. Features are then ranked by how much better they are at predicting the target variable compared to the shadow features. Those that don't perform better than random permutations are progressively eliminated. Because it uses a random forest (an ensemble method known for its robustness to overfitting), Boruta tends to be quite robust. By comparing each feature's importance with randomized features, Boruta ensures that only genuinely important features for predictive modeling are retained [40].

These methods automatically decide the optimal set of features based on the data and the problem at hand. Feature selection methods were applied to the radiomic features twice, the first time on the robust features (selected from the phantom study) and all 105 radiomic features extracted from each ROI of the clinical data set.

### Machine learning classifiers

Multiple machine learning classifiers were utilized for the prediction of LVI. We employed five machine learning algorithms: Logistic Regression (LR), XGBoost (XGB), Multilayer Perceptron (MLP), Naive Bayes (NB), and Random Forest (RF). All feature selection processes, and machine learning classification pipelines were implemented using an in-house developed tool based on the scikit-learn library in Python 3.9.12. We employed a 5-fold nested cross-validation approach for hyperparameter tuning of each model. In this methodology, the outer loop was responsible for splitting the dataset into training and test sets, while the inner loop performed model selection through hyperparameter tuning on the training set. The model selected by the inner loop was then evaluated on the test set provided by the outer loop. This process was repeated five times, ensuring that each fold was used exactly once as the test set. Each model pipeline consisted of a feature selector, and a machine learning classifier. To assess the stability and generalizability of each model, we applied 1000 bootstrap resampling in conjunction with the nested cross-validation. The models were evaluated based on various performance metrics, including Accuracy (ACC), Area Under the Curve (AUC), Sensitivity (SEN), Specificity (SPE), Negative Predictive Value (NPV), and Positive Predictive Value (PPV). Evaluations were conducted both with and without consideration of robustness of radiomic features to provide a comprehensive understanding of model performance.

### Statistical analysis

A Wilcoxon Rank-Sum test for *p*-value [41] was also implemented in R version 4.0.4 to calculate the p-value between the results of each machine learning classifier, feature selection algorithms, with and without considering the robustness of radiomic features. This statistical analysis was implemented to quantify the difference between the results, including ACC, AUC, and sensitivity of LVI prediction, with a 95% confidence interval. Differences with more than 95% confidence were categorized as significant.

## Results

**Fig 3:**
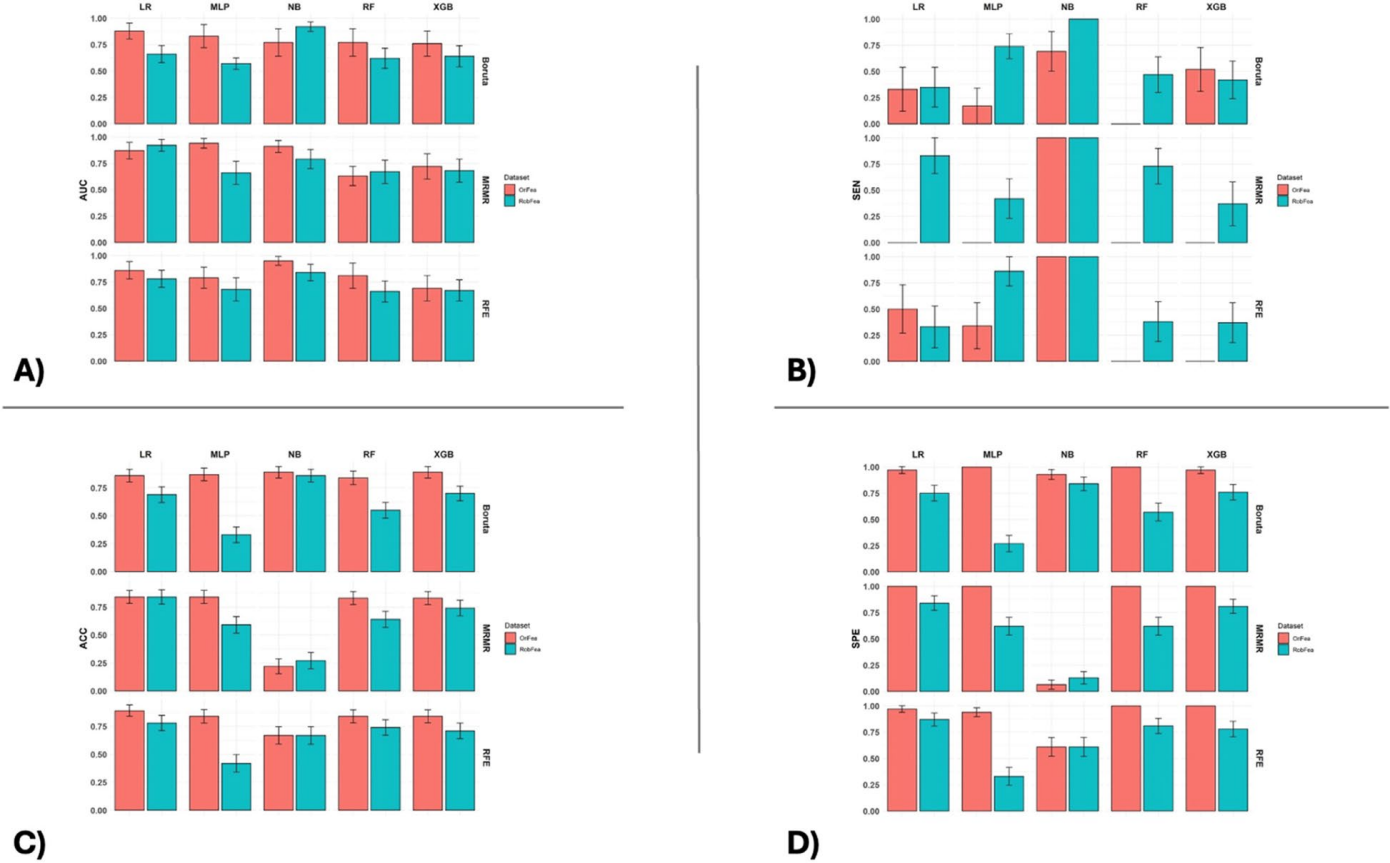
AUC (**A**), ACC (**B**), Sensitivity (**C**), Specificity (**D**) bar charts comparing the results of LVI prediction using multiple machine learning classifiers and various feature selection algorithms, with (RobFea) and without (OriFea) considering the robustness of radiomic features.

The results section of the current study consists of the ICC of 105 radiomic features extracted from phantom images, comparing the output of multiple machine learning classifiers and various feature selection algorithms belonging to all 105 radiomic features and robust features against the motion extracted from the clinical data set, including AUC, ACC, and sensitivity (Table 2). Figure 3 presents the heatmap of the AUC, ACC, and sensitivity of the LVI prediction. The final results are the outcome of the Wilcoxon Rank-Sum test for the p-value between the ACC, AUC, and sensitivity of the LVI prediction.

**Table 2:** ICC results, upper bound (Ubound), and lower bound (Lbound) with a 95% confidence interval belonging to the 24 robust features in the current study. Radiomic features were categorized based on their ICC into four groups, comprising 1) 90%≤ICC<100%, 2) 75%≤ICC<90%, 3) 50%≤ICC<75%, and 4) ICC<50%. Radiomic features with more than 75% ICC were selected as robust features and shown in this Table.

**Name**	**ICC**	**Lbound**	**Ubound**	**ICC group**
original_glrlm_RunLengthNonUniformity	0.983	0.844	0.998	1
original_shape_SurfaceVolumeRatio	0.982	0.866	0.998	1
original_shape_MeshVolume	0.977	0.835	0.997	1
original_shape_VoxelVolume	0.977	0.834	0.997	1
original_ngtdm_Coarseness	0.955	0.432	0.995	1
original_gldm_SmallDependenceLowGrayLevelEmphasis	0.950	0.569	0.994	1
original_shape_SurfaceArea	0.947	0.209	0.994	1
original_gldm_DependenceNonUniformity	0.945	0.360	0.994	1
original_shape_LeastAxisLength	0.941	0.571	0.993	1
original_firstorder_10Percentile	0.928	0.459	0.992	1
original_shape_Maximum2DDiameterSlice	0.928	0.336	0.992	1
original_glcm_MCC	0.921	0.425	0.991	1
original_gldm_DependenceEntropy	0.899	-0.135	0.990	2
original_firstorder_Kurtosis	0.896	0.074	0.989	2
original_shape_MinorAxisLength	0.891	0.218	0.988	2
original_firstorder_Minimum	0.879	0.067	0.987	2
original_glcm_Correlation	0.872	0.044	0.986	2
original_glszm_GrayLevelNonUniformity	0.855	-0.001	0.984	2
original_glszm_SizeZoneNonUniformity	0.830	-0.292	0.982	2
original_gldm_LargeDependenceHighGrayLevelEmphasis	0.828	-0.289	0.982	2
original_glszm_ZoneEntropy	0.820	-0.214	0.980	2
original_firstorder_Skewness	0.814	-0.208	0.979	2
original_firstorder_Median	0.778	-0.112	0.976	2
original_glcm_Idmn	0.759	-0.317	0.973	2

Table 2 indicates the upper bound (Ubound), lower bound (Lbound) with a 95% confidence interval, and the results of the ICC test to categorize Radiomic features based on their ICC against the lung motion into four groups, comprising 1) 90%≤ICC<100%, 2) 75%≤ICC<90%, 3) 50%≤ICC<75%, and 4) ICC<50%. Radiomic features with more than 75% ICC were selected as robust features.

The AUC bar charts shown in Figure 3A compare the AUC of the LVI prediction using different machine learning classifiers and feature selection algorithms with and without considering the robustness of radiomic features. Figure 3A demonstrates that, although the use of robust features by feature selection algorithms predominantly decreases the AUC values for predicting LVI with different machine learning classifiers, the extent of this decrease is not significant enough to warrant concern. Essentially, while there is a negative impact observed in the majority of the results (12 out of 15), the actual reduction in predictive accuracy (measured by AUC) is minor. This suggests that the drop in performance, though noticeable, does not critically impair the effectiveness of the classifiers when using robust features for feature selection. Thus, the trade-off between using robust features for improved generalizability and a slight decrease in predictive power might still be acceptable in practical applications, and in 3 outcomes, such as LR classifiers with MRMR feature selection (87% to 92%), NB classifiers with Boruta feature selection (77% to 92%), RF classifiers with MRMR feature selection (63% to 67%), the AUC of LVI prediction belonging to robust features was higher than the common methods without considering the robustness of radiomic features. The highest negative impact of selecting robust features on the AUC of prediction was achieved by the MLP classifier where the AUC decreased from 94% to 66% and from 83% to 57% for MRMR and Boruta feature selection, respectively.

Figure 3B presents the ACC bar charts of LVI prediction using various machine learning classifiers and feature selection algorithms with and without considering the robustness of radiomic features. Similarly with the AUC, using robust features adversely affects the ACC for the most parts (12 of 15 results). The ACC of the LR classifier with MRMR feature selection and NB classifier with RFE feature selection remained constant at 87% and 64%, respectively. Moreover, the ACC of the NB classifier with MRMR feature selection raised gently from 22% to 27%. Likewise, the largest adverse influence of selecting robust features on the ACC of prediction, was achieved by the MLP classifier, where the AUC decreased from 87% to 33% and from 84% to 59% for the Boruta and MRMR feature selection, respectively.

Figure 3C illustrates the sensitivity bar charts of LVI prediction using various machine learning classifiers and feature selection algorithms with and without considering the robustness of radiomic features. The greatest impact of using robust features as input to feature selection methods is in the sensitivity of prediction (Figure 3C). The sensitivity of LVI prediction after selecting robust features surged in the majority of outcomes (13 of 15). According to Figure 2C, using robust features boosted the sensitivity of prediction from zero to more than 83% in the LR classifier with MRMR feature selection. In the MLP classifier with MRMR feature selection (0 to 42%), the RF classifier with three feature selections implemented in this study (Boruta: 0 to 47%, MRMR: 0 to 73%, RFE: 0 to 38%), and the XGB classifier with MRMR (0 to 37%) and RFE (0 to 37%) feature selection, the sensitivity improved to a large extent. Moreover, although there are seven zeros in the sensitivity results of the original features, none of the predictions using robust features is equal to zero. The sensitivity of the NB classifier with Boruta feature selection raised from 69% to 100% as well.

As depicted in Figure 3D, the bar charts display the specificity of LVI prediction across different machine learning classifiers and feature selection algorithms, both with and without the incorporation of robust radiomic features. Notably, Figure 3D reveals that the specificity metrics experienced a decline across all models when robust features were utilized.

Out of 24 robust features, the following features were selected by between one and three feature selection algorithms conducted in the current study. Robust features are arranged by prevalence of selection: original_glrlm_RunLengthNonUniformity (selected by MRMR, RFE, Boruta), original_shape_MeshVolume (selected by MRMR, RFE, Boruta), original_ngtdm_Coarseness (selected by MRMR, RFE, Boruta), original_shape_SurfaceArea (selected by MRMR, RFE, Boruta), original_firstorder_10Percentile (selected by MRMR, RFE), original_gldm_DependenceEntropy (selected by MRMR, RFE), original_glcm_Correlation (selected by MRMR, Boruta), original_glszm_GrayLevelNonUniformity (selected by RFE, Boruta), original_firstorder_Skewness (selected by RFE, Boruta), and original_glcm_Idmn (selected by MRMR).

Contrarily, only 3 robust features were selected by the feature selection when all features were inputted, including original_ngtdm_Coarseness (selected by MRMR, RFE, Boruta), original_glszm_GrayLevelNonUniformity (selected by RFE, Boruta), and original_glcm_Idmn (selected by MRMR). The remaining selected features were not among the robust features, which indicates the inability of feature selection models in terms of taking into account of the robustness of radiomic features and raises concerns regarding the reproducibility of radiomics analysis.

**Fig 4:**
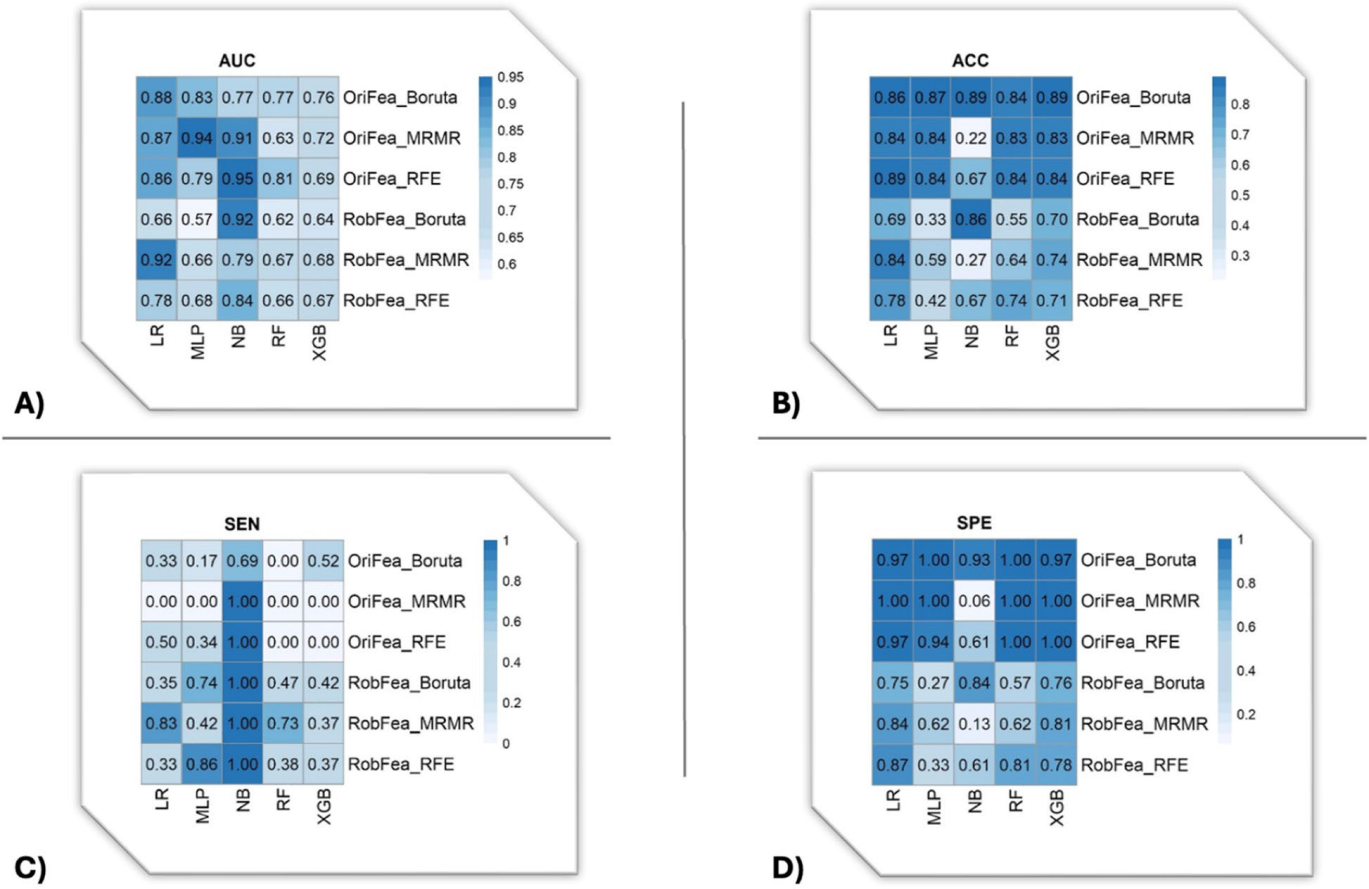
The AUC (**A**), ACC (**B**), sensitivity (**C**), and Specificity (**D**) heatmaps of LVI prediction using multiple machine learning classifiers and various feature selection algorithms, with (RobFea) and without (OriFea) considering the robustness of radiomic features.

Figure 4 depicts the heatmap of AUC (A), ACC (B), and sensitivity (C) of the LVI prediction. The highest AUC (95%) is associated with the NB classifier and RFE feature selection, followed by the MLP classifier and MRMR feature selection with 94% AUC without considering the robustness of radiomic features. After using robust features, the LR classifier with MRMR feature selection and NB classifier with Boruta feature selection demonstrated the highest AUC (92%). The highest ACC (89%) belonged to the LR classifier with RFE feature selection and XGB with Boruta feature selection, without considering the robustness of radiomic features. After using robust features, the NB classifier and Boruta feature selection resulted in the highest ACC (86%). In terms of sensitivity, the NB classifier showed that considering results after selecting robust features by feature selection methods resulted in 100%. However, RFE and MRMR feature selection with the NB classifier resulted in 100% sensitivity without considering the robustness of radiomic features.

Table 3 represents the mean standard deviation with a 95% confidence interval of the results of multiple machine learning classifiers and various feature selection algorithms, with and without considering the robustness of radiomic features. Table 4 shows the confidence interval of these results as well.

**Table 3:** The mean standard deviation (SD) of 1000 bootstraps belonged to the results of multiple machine learning classifiers and various feature selection algorithms, with (RobFea) and without (OriFea) considering the robustness of radiomic features.

**Type**	**AUC**	**ACC**	**SEN**	**SPE**	**PPV**	**NPV**
OriFea_Boruta_LR	0.88 ± 0.07	0.86 ± 0.05	0.33 ± 0.21	0.97 ± 0.03	0.66 ± 0.32	0.88 ± 0.05
OriFea_Boruta_NB	0.77 ± 0.13	0.89 ± 0.05	0.69 ± 0.19	0.93 ± 0.04	0.68 ± 0.2	0.94 ± 0.04
OriFea_Boruta_XGB	0.76 ± 0.12	0.89 ± 0.05	0.52 ± 0.21	0.97 ± 0.03	0.76 ± 0.24	0.91 ± 0.05
OriFea_Boruta_RF	0.77 ± 0.13	0.84 ± 0.06	0.00 ± 0.00	1.00 ± 0.00	0.93 ± 0.25	0.84 ± 0.06
OriFea_Boruta_MLP	0.83 ± 0.11	0.87 ± 0.05	0.17 ± 0.17	1.00 ± 0.00	0.96 ± 0.19	0.86 ± 0.05
OriFea_MRMR_LR	0.87 ± 0.07	0.84 ± 0.05	0.00 ± 0.00	1.00 ± 0.00	0.36 ± 0.48	0.84 ± 0.05
OriFea_MRMR_NB	0.91 ± 0.05	0.22 ± 0.06	1.00 ± 0.00	0.06 ± 0.04	0.17 ± 0.06	1 ± 0
OriFea_MRMR_XGB	0.72 ± 0.12	0.83 ± 0.05	0.00 ± 0.00	1.00 ± 0.00	0.92 ± 0.27	0.83 ± 0.05
OriFea_MRMR_RF	0.63 ± 0.09	0.83 ± 0.05	0.00 ± 0.00	1.00 ± 0.00	0.76 ± 0.43	0.83 ± 0.05
OriFea_MRMR_MLP	0.94 ± 0.04	0.84 ± 0.05	0.00 ± 0.00	1.00 ± 0.00	0.37 ± 0.48	0.84 ± 0.05
OriFea_RFE_LR	0.86 ± 0.08	0.89 ± 0.05	0.5 ± 0.23	0.97 ± 0.03	0.74 ± 0.25	0.91 ± 0.05
OriFea_RFE_NB	0.95 ± 0.04	0.67 ± 0.07	1.00 ± 0.00	0.61 ± 0.08	0.33 ± 0.11	1 ± 0
OriFea_RFE_XGB	0.69 ± 0.12	0.84 ± 0.05	0.00 ± 0.00	1.00 ± 0.00	0.93 ± 0.26	0.84 ± 0.05
OriFea_RFE_RF	0.81 ± 0.12	0.84 ± 0.05	0.00 ± 0.00	1.00 ± 0.00	0.76 ± 0.43	0.84 ± 0.05
OriFea_RFE_MLP	0.79 ± 0.1	0.84 ± 0.06	0.34 ± 0.22	0.94 ± 0.04	0.5 ± 0.28	0.88 ± 0.05
RobFea_Boruta_LR	0.66 ± 0.08	0.69 ± 0.07	0.35 ± 0.19	0.75 ± 0.07	0.21 ± 0.13	0.86 ± 0.06
RobFea_Boruta_NB	0.92 ± 0.04	0.86 ± 0.05	1.00 ± 0.00	0.84 ± 0.06	0.54 ± 0.16	1 ± 0
RobFea_Boruta_XGB	0.64 ± 0.1	0.7 ± 0.06	0.42 ± 0.18	0.76 ± 0.07	0.24 ± 0.11	0.87 ± 0.06
RobFea_Boruta_RF	0.62 ± 0.09	0.55 ± 0.07	0.47 ± 0.17	0.57 ± 0.08	0.17 ± 0.07	0.85 ± 0.07
RobFea_Boruta_MLP	0.57 ± 0.05	0.33 ± 0.07	0.74 ± 0.12	0.27 ± 0.07	0.14 ± 0.05	0.85 ± 0.08
RobFea_MRMR_LR	0.92 ± 0.05	0.84 ± 0.06	0.83 ± 0.17	0.84 ± 0.07	0.5 ± 0.16	0.96 ± 0.03
RobFea_MRMR_NB	0.79 ± 0.09	0.27 ± 0.07	1 ± 0	0.13 ± 0.06	0.18 ± 0.06	1 ± 0
RobFea_MRMR_XGB	0.68 ± 0.11	0.74 ± 0.07	0.37 ± 0.21	0.81 ± 0.06	0.27 ± 0.15	0.87 ± 0.06
RobFea_MRMR_RF	0.67 ± 0.11	0.64 ± 0.07	0.73 ± 0.17	0.62 ± 0.08	0.27 ± 0.1	0.92 ± 0.05
RobFea_MRMR_MLP	0.66 ± 0.11	0.59 ± 0.07	0.42 ± 0.19	0.62 ± 0.08	0.17 ± 0.08	0.84 ± 0.07
RobFea_RFE_LR	0.78 ± 0.08	0.78 ± 0.06	0.33 ± 0.2	0.87 ± 0.06	0.34 ± 0.21	0.87 ± 0.05
RobFea_RFE_NB	0.84 ± 0.07	0.67 ± 0.07	1.00 ± 0.00	0.61 ± 0.09	0.34 ± 0.11	1 ± 0
RobFea_RFE_XGB	0.67 ± 0.1	0.71 ± 0.07	0.37 ± 0.19	0.78 ± 0.07	0.25 ± 0.14	0.86 ± 0.06
RobFea_RFE_RF	0.66 ± 0.1	0.74 ± 0.06	0.38 ± 0.19	0.81 ± 0.07	0.29 ± 0.15	0.86 ± 0.06
RobFea_RFE_MLP	0.68 ± 0.11	0.42 ± 0.07	0.86 ± 0.14	0.33 ± 0.08	0.2 ± 0.07	0.92 ± 0.08

**Table 4:** The confidence interval (CI) of 1000 bootstraps for multiple machine learning classifiers and various feature selection algorithms, with (RobFea) and without (OriFea) considering the robustness of radiomic features.

**Type**	**AUC**	**ACC**	**SEN**	**SPE**	**PPV**	**NPV**
OriFea_Boruta_LR	0.88 - 0.89	0.86 - 0.87	0.32 - 0.34	0.96 - 0.97	0.64 - 0.68	0.88 - 0.89
OriFea_Boruta_NB	0.77 - 0.78	0.89 - 0.90	0.67 - 0.7	0.93 - 0.94	0.66 - 0.69	0.94 - 0.94
OriFea_Boruta_XGB	0.75 - 0.77	0.89 - 0.90	0.51 - 0.53	0.97 - 0.97	0.74 - 0.77	0.91 - 0.91
OriFea_Boruta_RF	0.76 - 0.78	0.83 - 0.84	0.00 – 0.00	1.00 – 1.00	0.92 - 0.95	0.83 - 0.84
OriFea_Boruta_MLP	0.82 - 0.83	0.86 - 0.87	0.16 - 0.18	1.00 – 1.00	0.95 - 0.98	0.86 - 0.87
OriFea_MRMR_LR	0.87 - 0.88	0.83 - 0.84	0.00 – 0.00	1.00 – 1.00	0.33 - 0.39	0.83 - 0.84
OriFea_MRMR_NB	0.90 - 0.91	0.21 - 0.22	1.00 – 1.00	0.061 - 0.067	0.17 - 0.18	1.00 – 1.00
OriFea_MRMR_XGB	0.71 - 0.73	0.83 - 0.84	0.00 – 0.00	1.00 – 1.00	0.9 - 0.94	0.83 - 0.84
OriFea_MRMR_RF	0.62 - 0.63	0.83 - 0.84	0.00 – 0.00	1.00 – 1.00	0.73 - 0.79	0.83 - 0.84
OriFea_MRMR_MLP	0.93 - 0.94	0.83 - 0.84	0.00 – 0.00	1.00 – 1.00	0.34 - 0.4	0.83 - 0.84
OriFea_RFE_LR	0.85 - 0.86	0.89 - 0.89	0.48 - 0.51	0.97 - 0.97	0.72 - 0.75	0.91 - 0.91
OriFea_RFE_NB	0.95 - 0.96	0.67 - 0.68	1.00 – 1.00	0.6 - 0.61	0.32 - 0.34	1.00 – 1.00
OriFea_RFE_XGB	0.68 - 0.70	0.83 - 0.84	0.00 – 0.00	1.00 – 1.00	0.91 - 0.95	0.83 - 0.84
OriFea_RFE_RF	0.81 - 0.82	0.83 - 0.84	0.00 – 0.00	1.00 – 1.00	0.74 - 0.79	0.83 - 0.84
OriFea_RFE_MLP	0.79 - 0.8	0.83 - 0.84	0.33 - 0.35	0.93 - 0.94	0.48 - 0.51	0.87 - 0.88
RobFea_Boruta_LR	0.66 - 0.67	0.68 - 0.69	0.34 - 0.37	0.75 - 0.76	0.21 - 0.22	0.86 - 0.86
RobFea_Boruta_NB	0.92 - 0.92	0.86 - 0.87	1.00 – 1.00	0.83 - 0.84	0.53 - 0.55	1.00 – 1.00
RobFea_Boruta_XGB	0.63 - 0.65	0.70 - 0.71	0.41 - 0.44	0.75 - 0.76	0.24 - 0.25	0.87 - 0.88
RobFea_Boruta_RF	0.62 - 0.63	0.54 - 0.56	0.46 - 0.49	0.56 - 0.58	0.16 - 0.17	0.84 - 0.86
RobFea_Boruta_MLP	0.55 - 0.58	0.31 - 0.35	0.7 - 0.77	0.25 - 0.29	0.12 - 0.15	0.83 - 0.87
RobFea_MRMR_LR	0.92 - 0.92	0.83 - 0.84	0.82 - 0.84	0.83 - 0.84	0.49 - 0.51	0.96 - 0.96
RobFea_MRMR_NB	0.78 - 0.79	0.26 - 0.27	1.00 – 1.00	0.13 - 0.13	0.18 - 0.19	1.00 – 1.00
RobFea_MRMR_XGB	0.68 - 0.69	0.73 - 0.74	0.35 - 0.38	0.81 - 0.82	0.26 - 0.28	0.86 - 0.87
RobFea_MRMR_RF	0.66 - 0.68	0.63 - 0.64	0.72 - 0.74	0.61 - 0.63	0.26 - 0.28	0.91 - 0.92
RobFea_MRMR_MLP	0.65 - 0.67	0.58 - 0.59	0.40 - 0.43	0.61 - 0.63	0.16 - 0.18	0.84 - 0.85
RobFea_RFE_LR	0.78 - 0.79	0.78 - 0.78	0.32 - 0.35	0.86 - 0.87	0.32 - 0.35	0.87 - 0.87
RobFea_RFE_NB	0.84 - 0.85	0.67 - 0.68	1.00 – 1.00	0.6 - 0.61	0.33 - 0.34	1.00 – 1.00
RobFea_RFE_XGB	0.66 - 0.68	0.71 - 0.72	0.36 - 0.38	0.78 - 0.79	0.24 - 0.26	0.85 - 0.86
RobFea_RFE_RF	0.65 - 0.67	0.73 - 0.74	0.36 - 0.39	0.81 - 0.82	0.28 - 0.3	0.86 - 0.87
RobFea_RFE_MLP	0.67 - 0.69	0.41 - 0.42	0.85 - 0.86	0.32 - 0.33	0.20 - 0.21	0.91 - 0.92

**Fig 5:**
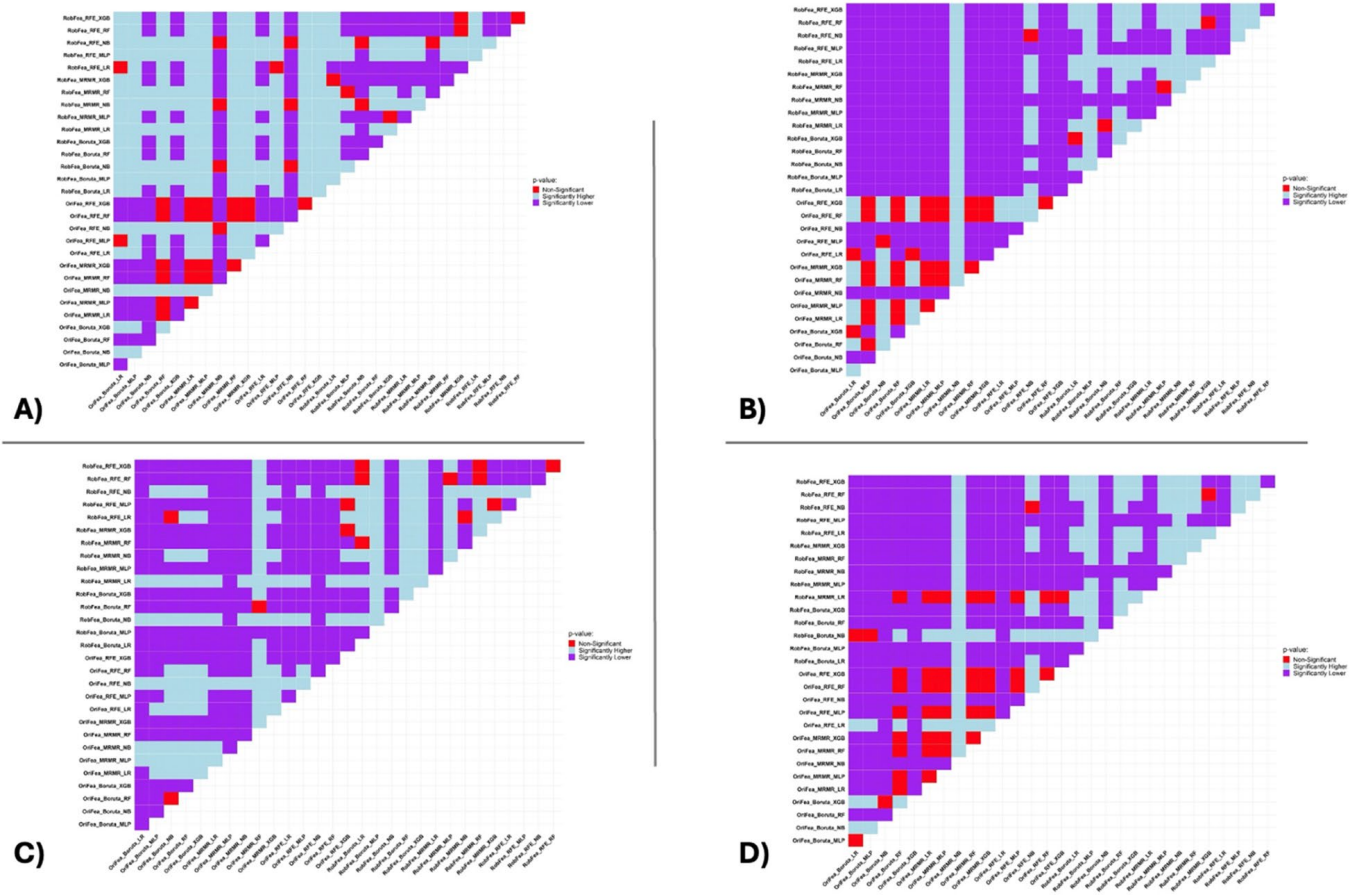
Wilcoxon Rank-Sum test for *p*-value results for the AUC (**A**), ACC (**B**), sensitivity (**C**), specificity (**D**) of LVI prediction using multiple machine learning classifiers and various feature selection algorithms, with (RobFea) and without (OriFea) considering the robustness of radiomic features

According to Figure 5A 3% (14 of 465) of the Wilcoxon Rank-Sum test for p-value results for the AUC of the LVI prediction were non-significant, including original features of Boruta feature selection with RF classifier and Boruta feature selection with NB classifier. Sixty-two percent of the results were significantly lower, which indicated that using robust features adversely affects the AUC of prediction by more than 5%. According to figure 5B, the number of non-significant ACC results of the Wilcoxon Rank-Sum test for the p-value test is higher than AUC (42 out of 465 equals 9%). Seventy-four percent of the results were significantly lower, which shows that using robust features as input to feature selection mostly has a negative impact on the ACC results of prediction. Figure 5C indicates that the number of significantly higher results of the Wilcoxon Rank-Sum test for the p-value test is considerably higher than AUC and ACC, where 32% and 11% of the results were significantly lower and non-significant, respectively. According to Figure 5D, 66% and 16% of the specificity results were significantly lower and non-significant, respectively.

## Discussion

Radiomics, an emerging tool to extract hidden information from medical images, has been widely utilized in previous studies. The application of radiomics encompasses predicting response to therapy [42], and predicting and diagnosing abnormities [43]. However, radiomic features are vulnerable to various factors, including motion [44] and multi-center studies [45]. Various strategies were devised to overcome the low repeatability and reproducibility of radiomic features, such as selecting robust features against the influential factor and Combat harmonization. Even though the selection of robust features was examined in previous studies, the number of studies that studied the impact of motion on radiomic features, especially on PET images, is relatively small. In few studies, the effect of lung movement on radiomic features was evaluated [13]. Lung movement has a considerable impact on radiomic features, where 11% and 12% of them showed ICC of more than 90% and 75%≤ICC<90%, respectively, and were considered robust features against motion (24 out of 105 feature). Even though feature selection algorithms are powerful at choosing bold features that boost exploring data and identifying patterns, they might have weaknesses in considering the repeatability and reproducibility of radiomic features.

In the current study, we aimed at selecting robust features against lung motion in a phantom study followed by using these robust features as input to various feature selection methods and comparing these results with conventional techniques without considering the reproducibility of radiomic features to assess the impact of considering the robustness of radiomic features on the results of machine learning classifiers.

In a recent PET-based radiomics study, Hu et al. [46] predicted lymph node metastasis for 794 NSCLC patients using machine learning classifiers and natural language processing, resulting in 79% AUC with random forest classifiers. In our results, by comparison, the RF and NB classifiers with RFE feature selection of original features achieved 81% and 95% AUC, respectively. In a CT-based radiomics analysis, Peng et al. [47] examined the potential of radiomic features to predict LVI in esophageal squamous cell carcinoma using different machine learning algorithms. According to their results involving 294 patients, the highest reported AUC was 79% and 66% for the training cohort and validation cohort, respectively. In a machine learning PET/CT study, Fan et al. [48] predicted LVI using clinical factors, PET radiomics, CT radiomics, and their combination with three machine learning classifiers, such as adaptive boosting (AdaBoost), LR, and linear discriminant analysis (LDA). Based on their results, the best performance in terms of AUC was achieved by the combined model, image model, and clinical factors with 94%, 84%, and 74% AUC of the AdaBoost classifier. None of these studies considered the repeatability of radiomic features, and radiomic features were selected by feature selection algorithms.

There have been a few studies that considered the robustness of radiomic features in a machine/deep learning approach. Mostafa et al. [49] aimed to identify robust radiomic features from ^18^F-FDG PET/CT images of NSCLC patients, assess their reproducibility across different segmentation methods, and evaluate their prognostic value for 2-year overall survival. Employing four distinct segmentation techniques, the authors found 10 robust radiomic features, with three texture features showing association with 2-year overall survival. Comparing these results to our findings, there are notable parallels and distinctions in the pursuit of robust radiomic features in NSCLC LVI prediction. Both studies underscore the critical importance of feature robustness—ours through the lens of motion artifact resilience and theirs through segmentation method consistency. Our study identified a set of robust features consistently selected by multiple feature selection algorithms, highlighting their potential predictive power and stability, similar to the emphasis on features' concordance correlation coefficient in the summarized study. In both studies, the feature Original_glszm_GrayLevelNonUniformity was identified as robust and bold feature and was selected by RFE and Boruta algorithms in our analysis. Pasini et al. [50] delved into the challenges and potential of radiomics in classifying the histopathological subtypes of NSCLC by extracting 1781 radiomics features from multicenter CT images across four NSCLC subtypes using IBSI-compliant tool. They investigated batch effects, feature harmonization's impact on model performance, and how training dataset composition affects feature selection and model accuracy. The emphasized importance of texture features and the challenges in multiclass classification resonate with our findings, suggesting that both studies contribute to a growing understanding of the use of radiomics in cancer classification and prognosis. Tanaka et al. [51], in an interesting deep learning radiomics study, predicted head and neck tumor regression. They extracted radiomic features with a deep learning method and selected robust features with ICC>0.7 against various segmentation methods, followed by using them as input of feature selection algorithms. Next, five feature selection algorithms and five machine learning classifiers were used, including RF, KNN, LDA, NB, and SVM with 1000 bootstrap. The highest reported AUC, according to their results, was 84%.

The use of robust features in our study yielded an interesting shift in performance metrics. Specifically, we observed an increase in sensitivity at the cost of a slight reduction in accuracy and AUC. The incorporation of robust features is likely to contribute to this phenomenon due to their resilience against motion artifacts in the phantom study, which could be particularly effective in capturing the nuances required to correctly identify positive LVI cases, thus improving sensitivity. In the medical context, high sensitivity is often desirable, as it reduces the likelihood of false negatives, which is critical in cancer diagnosis and prognosis [52]. However, robust features are, by design, less sensitive to variations in data that may not be due to artifacts. This could mean that while they are excellent for detecting true positive cases (thereby increasing sensitivity), they might be less adept at distinguishing between true negatives and false positives, which could explain the observed decrease in specificity and, subsequently, accuracy and AUC. Additionally, feature selection algorithms (RFE and Boruta) and machine learning classifiers (particularly Naive Bayes) likely interact differently with robust features. For example, Naive Bayes assumes independence between features, an assumption that may or may not hold with robust features [53]. The performance gains or losses observed could partly be due to how well these algorithms leverage the unique properties of robust features.

Although in a few radiomics-based studies, the repeatability and reproducibility of radiomic features have been evaluated, there is a lack of studies using robust features against lung motion from a phantom study and utilizing them through machine/deep learning evaluation. To the best of our knowledge, there is no similar study comparing the results of multiple machine learning algorithms and various feature selection methods with and without considering the robustness of radiomics features for the same problem. Furthermore, in line with IBSI guidelines [32, 33], it is advisable to employ radiomic features that exhibit high repeatability and reproducibility for more reliable and consistent outcome prediction.

The selection of robust features in the prediction of LVI in NSCLC underscores a pivotal advancement in enhancing the accuracy and reliability of radiomics-based prognostic models. The identification of a core set of radiomic features, such as original_glrlm_RunLengthNonUniformity, original_shape_MeshVolume, and original_ngtdm_Coarseness by multiple feature selection algorithms (MRMR, RFE, Boruta) highlights their potential robustness and predictive power in the context of NSCLC LVI prediction. These features, selected consistently across different algorithms, signify a critical intersection between mathematical robustness and clinical relevance, suggesting that they capture fundamental aspects of tumor biology that are minimally affected by external variabilities, such as imaging technique or patient movement. This consistency not only reinforces the validity of these features as biomarkers for LVI but also reflects on the sophistication of feature selection methodologies in distinguishing the most predictive and stable features amidst a plethora of radiomic data.

Conversely, the finding that only a limited number of robust features were selected when all features were inputted into the selection algorithms raises concerns about the current capabilities of these methodologies to fully appreciate the importance of feature robustness. This observation suggests a potential gap in the feature selection process, where algorithms may prioritize statistical relevance over clinical utility and reproducibility. The reliance on non-robust features could lead to models that perform well in controlled or specific datasets but fail to generalize across different clinical settings or imaging protocols. This issue highlights a critical area for future research and development in radiomics: the need for feature selection algorithms that are inherently designed to consider the robustness of features, ensuring that the selected features are not only predictive but also reliably reproducible across diverse clinical conditions. Addressing this challenge is essential for the progress of radiomics from a research tool to a standard component of precision oncology in NSCLC, particularly for critical prognostic indicators, such as LVI.

Phantom studies allow for controlled and reproducible motion simulations, ensuring that the impact of motion on radiomic features can be systematically analyzed without biological variability. Unlike patient studies, phantom studies provide ground-truth measurements, eliminating inter-subject anatomical variations and ensuring that the observed effects are purely due to motion rather than other confounding factors.

Our study inherently bears some limitations. The sample size used in our study set is the foremost limitation and future studies with a larger dataset is needed to validate these results and investigate hybrid feature selection approaches that integrate multiple selection strategies to enhance model robustness and performance. Radiomic features are vulnerable against various factors. Other effective factors, specially multi center data should also be examined in future studies. The use of robust features in deep learning or deep radiomics was not considered in the current study and will be evaluated in future studies. Future studies will explore alternative methods, including retrospective correction techniques or motion-gated acquisitions, to complement our current findings. Additionally, this study considered only two-dimensional motion and a single tumor size. Future studies should explore three-dimensional motion dynamics and different lesion sizes to better reflect clinical scenarios.

## Conclusion

Our study underscores the benefits and trade-offs of employing robust features for LVI prediction in NSCLC. While the use of robust features improves the model's sensitivity, which is often crucial in medical applications, it does so at the expense of accuracy and AUC. Given the high stakes involved in accurate cancer diagnosis and treatment, the increase in sensitivity might be clinically more valuable, even if it comes at the cost of other performance metrics. Therefore, the adoption of robust features could be a promising avenue for future research, particularly for applications where high sensitivity is a priority.

## Data Availability

No datasets were generated or analysed during the current study.

## References

[1] Prabhakar B, Shende P, Augustine S. Current trends and emerging diagnostic techniques for lung cancer. Biomedicine & Pharmacotherapy. 2018;106:1586–99. 30119234 10.1016/j.biopha.2018.07.145

[2] Bade BC, Cruz CSD. Lung cancer 2020: epidemiology, etiology, and prevention. Clinics in chest medicine. 2020;41:1–24. 32008623 10.1016/j.ccm.2019.10.001

[3] Siegel RL, Miller KD, Jemal A. Cancer statistics, 2018. CA: a cancer journal for clinicians. 2018;68:7-30. 10.3322/caac.21442 29313949

[4] BoshoffC HerbstRS M. TheBiologyandManagementofNon-Small Cell Lung Cancer. Nature. 2018;553:446–54. 29364287 10.1038/nature25183

[5] Sung SY, Kwak Y-K, Lee S-W, Jo IY, Park JK, Kim KS, et al. Lymphovascular Invasion Increases the Risk of Nodal and Distant Recurrence in Node-Negative Stage I-IIA Non-Small-Cell Lung Cancer. Oncology. 2018;95:156–62. 29847825 10.1159/000488859

[6] Mollberg NM, Bennette C, Howell E, Backhus L, Devine B, Ferguson MK. Lymphovascular invasion as a prognostic indicator in stage I non-small cell lung cancer: a systematic review and meta-analysis. The Annals of Thoracic Surgery. 2014;97:965–71. 24424014 10.1016/j.athoracsur.2013.11.002

[7] Kalemaki MS, Karantanas AH, Exarchos D, Detorakis ET, Zoras O, Marias K, et al. PET/CT and PET/MRI in ophthalmic oncology. International journal of oncology. 2020;56:417–29. 31939615 10.3892/ijo.2020.4955 PMC6959466

[8] Hosseini SA, Shiri I, Hajianfar G, Bagley S, Nasrallah M, O’Rourke DM, Mohan S, Chawla S, MRI based Radiomics for Distinguishing IDH-mutant from IDH wild-type Grade-4 Astrocytomas. In Proceedings of the 31st Annual Meeting of ISMRM, London, UK, 7–12 May 2022.

[9] Hatt M, Krizsan AK, Rahmim A, Bradshaw TJ, Costa PF, Forgacs A, et al. Joint EANM/SNMMI guideline on radiomics in nuclear medicine : Jointly supported by the EANM Physics Committee and the SNMMI Physics, Instrumentation and Data Sciences Council. Eur J Nucl Med Mol Imaging. 2023;50:352–75. 36326868 10.1007/s00259-022-06001-6 PMC9816255

[10] Hosseini SA, Hajianfar G, Shiri I, Zaidi H, Lung Cancer Recurrence Prediction Using Radiomics Features of PET Tumor Sub-Volumes and Multi-Machine Learning Algorithms. In Proceedings of the 2021 IEEE Nuclear Science Symposium and Medical Imaging Conference (NSS/MIC), Piscataway, NJ, USA, 16–23 October 2021.

[11] Hosseini SA, Hosseini E, Hajianfar G, Shiri I, Servaes S, Rosa-Neto P, et al. MRI-based radiomics combined with deep learning for distinguishing IDH-mutant WHO grade 4 astrocytomas from IDH-wild-type glioblastomas. Cancers. 2023;15:951. 36765908 10.3390/cancers15030951 PMC9913426

[12] Hajianfar G, Haddadi Avval A, Hosseini SA, Nazari M, Oveisi M, Shiri I, et al. Time-to-event overall survival prediction in glioblastoma multiforme patients using magnetic resonance imaging radiomics. La radiologia medica. 2023;128:1521–34. 37751102 10.1007/s11547-023-01725-3 PMC10700216

[13] Hosseini SA, Shiri I, Hajianfar G, Bahadorzadeh B, Ghafarian P, Zaidi H, et al. Synergistic impact of motion and acquisition/reconstruction parameters on 18F-FDG PET radiomic features in non-small cell lung cancer: Phantom and clinical studies. Medical Physics. 2022;49:3783–96. 35338722 10.1002/mp.15615 PMC9322423

[14] Hosseini SA, Hajianfar G, Shiri I, Zaidi H. PET Image Radiomics Feature Variability in Lung Cancer: Impact of Image Segmentation. In Proceedings of the 2021 IEEE Nuclear Science Symposium and Medical Imaging Conference (NSS/MIC), Piscataway, NJ, USA, 16–23 October 2021.

[15] Aide N, Lasnon C, Desmonts C, Armstrong IS, Walker MD, McGowan DR. Advances in PET/CT Technology: An Update. Semin Nucl Med. 2022;52:286–301. 34823841 10.1053/j.semnuclmed.2021.10.005

[16] Zaidi H, Karakatsanis N. Towards enhanced PET quantification in clinical oncology. Br J Radiol. 2018;91:20170508. 29164924 10.1259/bjr.20170508 PMC6049841

[17] Hosseini SA, Shiri I, Ghaffarian P, Hajianfar G, Avval AH, Seyfi M, et al. The effect of harmonization on the variability of PET radiomic features extracted using various segmentation methods. Ann Nucl Med. 2024;38(7):493–507. 10.1007/s12149-024-01923-7 PMC11217131 38575814

[18] Hajianfar G, Hosseini SA, Bagherieh S, Oveisi M, Shiri I, Zaidi H. Impact of harmonization on the reproducibility of MRI radiomic features when using different scanners, acquisition parameters, and image pre-processing techniques: A phantom study. Med Biol Eng Comput. 2024;62:2319–32. 10.1007/s11517-024-03071-6 PMC11604802 38536580

[19] Hosseini SA, Shiri I, Hajianfar G, Ghafarian P, Karam MB, Ay MR. The impact of preprocessing on the PET-CT radiomics features in non-small cell lung cancer. Frontiers in Biomedical Technologies. 2021;8:261–72.

[20] Hosseini SA, Hajianfar G, Hosseini E, Servaes S, Rosa-Neto P, Shiri I, et al. Robust versus Non-Robust Radiomic features: Machine Learning Based Models for NSCLC Lymphovascular Invasion. In Proceedings of the 2022 IEEE Nuclear Science Symposium and Medical Imaging Conference (NSS/MIC), Milan, Italy, 5–12 November 2022; pp. 1–3.

[21] Shiri I, Rahmim A, Ghaffarian P, Geramifar P, Abdollahi H, Bitarafan-Rajabi A. The impact of image reconstruction settings on 18F-FDG PET radiomic features: multi-scanner phantom and patient studies. European radiology. 2017;27:4498–509. 28567548 10.1007/s00330-017-4859-z

[22] Orlhac F, Frouin F, Nioche C, Ayache N, Buvat I. Validation of a method to compensate multicenter effects affecting CT radiomics. Radiology. 2019;291:53–9. 30694160 10.1148/radiol.2019182023

[23] Lu L, Lv W, Jiang J, Ma J, Feng Q, Rahmim A, et al. Robustness of radiomic features in [11 c] choline and [18 f] fdg pet/ct imaging of nasopharyngeal carcinoma: Impact of segmentation and discretization. Molecular Imaging and Biology. 2016;18:935–45. 27324369 10.1007/s11307-016-0973-6

[24] Lee J, Steinmann A, Ding Y, Lee H, Owens C, Wang J, et al. Radiomics feature robustness as measured using an MRI phantom. Scientific reports. 2021;11:3973. 33597610 10.1038/s41598-021-83593-3 PMC7889870

[25] Gómez OV, Herraiz JL, Udías JM, Haug A, Papp L, Cioni D, et al. Analysis of cross-combinations of feature selection and machine-learning classification methods based on [18F] F-FDG PET/CT radiomic features for metabolic response prediction of metastatic breast cancer lesions. Cancers. 2022;14:2922. 35740588 10.3390/cancers14122922 PMC9221062

[26] Demircioğlu A. Benchmarking feature selection methods in radiomics. Investigative radiology. 2022;57:433–43. 35045555 10.1097/RLI.0000000000000855

[27] Xue C, Yuan J, Lo GG, Poon DM, Chu WC. Evaluation of the Reliability and the Performance of Magnetic Resonance Imaging Radiomics in the Presence of Randomly Generated Irrelevant Features for Prostate Cancer. Diagnostics. 2023;13:3580. 38066821 10.3390/diagnostics13233580 PMC10705874

[28] Qadir MI, Muneer N. Coordination of Ladyfinger Likeliness and Normal Breathing Rate. Biomedical Journal of Scientific & Technical Research. 2019;15:11402–4.

[29] Xu Q, Yuan K, Ye D. Respiratory motion blur identification and reduction in ungated thoracic PET imaging. Physics in Medicine & Biology. 2011;56:4481. 21719945 10.1088/0031-9155/56/14/016

[30] Abdollahi B, Civelek AC, Li X-F, Suri J, El-Baz A. PET/CT nodule segmentation and diagnosis: A survey. Multi Detector CT Imaging. 2014:639-51.

[31] Zaidi H, Abdoli M, Fuentes CL, El Naqa IM. Comparative methods for PET image segmentation in pharyngolaryngeal squamous cell carcinoma. European journal of nuclear medicine and molecular imaging. 2012;39:881–91. 22289958 10.1007/s00259-011-2053-0 PMC3326239

[32] Zwanenburg A, Vallieres M, Abdalah MA, Aerts H, Andrearczyk V, Apte A, et al. The Image Biomarker Standardization Initiative: Standardized Quantitative Radiomics for High-Throughput Image-based Phenotyping. Radiology. 2020;295:328–38. 32154773 10.1148/radiol.2020191145 PMC7193906

[33] Whybra P, Zwanenburg A, Andrearczyk V, Schaer R, Apte AP, Ayotte A, et al. The image biomarker standardization initiative: Standardized convolutional filters for reproducible radiomics and enhanced clinical insights. Radiology. 2024;310: e231319. 38319168 10.1148/radiol.231319 PMC10902595

[34] Van Griethuysen JJ, Fedorov A, Parmar C, Hosny A, Aucoin N, Narayan V, et al. Computational radiomics system to decode the radiographic phenotype. Cancer research. 2017;77:e104-e07. 29092951 10.1158/0008-5472.CAN-17-0339 PMC5672828

[35] Shrout PE, Fleiss JL. Intraclass correlations: uses in assessing rater reliability. Psychological bulletin. 1979;86:420. 18839484 10.1037//0033-2909.86.2.420

[36] McGraw KO, Wong SP. Forming inferences about some intraclass correlation coefficients. Psychological methods. 1996;1:30.

[37] Bartko JJ. The intraclass correlation coefficient as a measure of reliability. Psychological reports. 1966;19:3–11. 5942109 10.2466/pr0.1966.19.1.3

[38] Mahmoud O, Harrison A, Perperoglou A, Gul A, Khan Z, Metodiev MV, et al. A feature selection method for classification within functional genomics experiments based on the proportional overlapping score. BMC bioinformatics. 2014;15:1–20. 25113817 10.1186/1471-2105-15-274 PMC4141116

[39] Molnar C, König G, Bischl B, Casalicchio G. Model-agnostic feature importance and effects with dependent features: a conditional subgroup approach. Data Min Knowl Discov. 2023:1-39. 10.1007/s10618-022-00901-9.

[40] Robust and Efficient Approach to Feature Selection With Machine Learning Faculty of Mathematics, Informatics and Mechanics. University of Warsaw; 2016.

[41] Perolat J, Couso I, Loquin K, Strauss O. Generalizing the Wilcoxon rank-sum test for interval data. International Journal of Approximate Reasoning. 2015;56:108–21.

[42] Castello A, Castellani M, Florimonte L, Urso L, Mansi L, Lopci E. The role of radiomics in the era of immune checkpoint inhibitors: a new protagonist in the jungle of response criteria. Journal of Clinical Medicine. 2022;11:1740. 35330068 10.3390/jcm11061740 PMC8948743

[43] Bera K, Braman N, Gupta A, Velcheti V, Madabhushi A. Predicting cancer outcomes with radiomics and artificial intelligence in radiology. Nature reviews Clinical oncology. 2022;19:132–46. 34663898 10.1038/s41571-021-00560-7 PMC9034765

[44] Adachi T, Nagasawa R, Nakamura M, Kakino R, Mizowaki T. Vulnerabilities of radiomic features to respiratory motion on four-dimensional computed tomography-based average intensity projection images: A phantom study. Journal of Applied Clinical Medical Physics. 2022;23:e13498. 35088515 10.1002/acm2.13498 PMC8906211

[45] Saltybaeva N, Tanadini-Lang S, Vuong D, Burgermeister S, Mayinger M, Bink A, et al. Robustness of radiomic features in magnetic resonance imaging for patients with glioblastoma: Multi-center study. Physics and imaging in radiation oncology. 2022;22:131–6. 35633866 10.1016/j.phro.2022.05.006 PMC9130546

[46] Hu D, Li S, Zhang H, Wu N, Lu X. Using Natural Language Processing and Machine Learning to Preoperatively Predict Lymph Node Metastasis for Non-Small Cell Lung Cancer With Electronic Medical Records: Development and Validation Study. JMIR Medical Informatics. 2022;10:e35475. 35468085 10.2196/35475 PMC9086872

[47] Peng H, Yang Q, Xue T, Chen Q, Li M, Duan S, et al. Computed tomography-based radiomics analysis to predict lymphovascular invasion in esophageal squamous cell carcinoma. The British Journal of Radiology. 2022;95:20210918. 34908477 10.1259/bjr.20210918 PMC8822548

[48] Fan L, Li J, Zhang H, Yin H, Zhang R, Zhang J, et al. Machine learning analysis for the noninvasive prediction of lymphovascular invasion in gastric cancer using PET/CT and enhanced CT-based radiomics and clinical variables. Abdominal Radiology. 2022;47:1209–22. 35089370 10.1007/s00261-021-03315-1

[49] Mostafa R, Kandeel AA, Abd Elkareem M, Nardo L, Abdelhafez YG. Pretherapy 18F-fluorodeoxyglucose positron emission tomography/computed tomography robust radiomic features predict overall survival in non-small cell lung cancer. Nuclear Medicine Communications. 2022;43:540–8. 35190518 10.1097/MNM.0000000000001541

[50] Pasini G, Stefano A, Russo G, Comelli A, Marinozzi F, Bini F. Phenotyping the histopathological subtypes of non-small-cell lung carcinoma: how beneficial is radiomics? Diagnostics. 2023;13:1167. 36980475 10.3390/diagnostics13061167 PMC10046953

[51] Tanaka S, Kadoya N, Sugai Y, Umeda M, Ishizawa M, Katsuta Y, et al. A deep learning-based radiomics approach to predict head and neck tumor regression for adaptive radiotherapy. Scientific Reports. 2022;12:8899. 35624113 10.1038/s41598-022-12170-z PMC9142601

[52] Schiffman JD, Fisher PG, Gibbs P. Early detection of cancer: past, present, and future. American Society of Clinical Oncology Educational Book. 2015;35:57–65. 10.14694/EdBook_AM.2015.35.57 25993143

[53] Rennie JD, Shih L, Teevan J, Karger DR. Tackling the poor assumptions of naive bayes text classifiers. Proceedings of the 20th international conference on machine learning (ICML-03). 2003.

